# Reply to “The SARS-COV Pandemic Does Not Absolve From Solid Medical Trade”

**DOI:** 10.31486/toj.21.5021

**Published:** 2021

**Authors:** Ivana Okor, Tamunoinemi Bob-Manuel, Selim R. Krim

**Affiliations:** ^1^ Department of Hospital Medicine, Ochsner Clinic Foundation, New Orleans, LA; ^2^ Department of Cardiology, John Ochsner Heart and Vascular Institute, Ochsner Clinic Foundation, New Orleans, LA; ^3^ The University of Queensland Faculty of Medicine, Ochsner Clinical School, New Orleans, LA

## TO THE EDITOR

We appreciate the response by Finsterer and Stöllberger to our case report “Suspected COVID-19–Induced Myopericarditis,”^1^ and we agree that our case has some limitations. We, however, hope to make some clarifications with this response to better explain our diagnostic and therapeutic decisions. We would prefix by stating that this case occurred very early in the COVID-19 pandemic before unifying guidelines, clinical trials, or approved therapies were available. During that time, our institution, like other institutions around the world, came up with specific guidelines on clinical practice to mitigate the spread of COVID-19 and improve clinical outcomes. These guidelines affected our choice of medications and standard of practice.

The policy at the time was to prophylactically treat for bacterial coinfection/superinfection in patients with COVID-19 pneumonia who were sick enough to be admitted to the hospital. One of the standard treatments for inpatient community-acquired pneumonia is an antipneumococcal beta-lactam (ceftriaxone) and a macrolide (azithromycin).^2^ Therefore, on admission to the hospital, the patient was treated with the 2 antibiotics ceftriaxone and azithromycin. Our patient never required mechanical ventilation due to COVID-19 pneumonia or acute respiratory distress syndrome. She was placed on a non-rebreather mask as needed for hypoxia, with SpO_2_ maintained above 92%.

On hospital day 4, the patient was admitted to the intensive care unit, primarily because of encephalopathy and clinical findings consistent with cardiogenic shock. Computed tomography of the head did not reveal any ischemia or intracranial hemorrhage at the initial discovery of altered mental status. The subsequent workup for acute encephalopathy involved evaluating for acute infection; tests included a chest x-ray, blood cultures, and urinalysis. However, the presence of low cardiac output state and acute electrocardiogram (ECG) changes, along with echocardiographic evidence of severely depressed left ventricular systolic function occurring concurrently with the acute mental status changes, pointed toward cardiogenic shock as the etiology of her encephalopathy.

We note that acute coronary syndrome, myopericarditis, and Takotsubo cardiomyopathy were on the differential. However, we had evidence pointing toward myopericarditis as the likely pathologic process. While invasive coronary angiography is indispensable to the workup, certain factors precluded the patient from getting one. First was her hemodynamic instability and concern for active infection. Second, the surge of the COVID-19 pandemic called for dynamic triage with definite obstructive disease prioritized for percutaneous coronary intervention. Of note, serial ECGs and cardiac enzyme trend were not consistent with an evolving myocardial infarction, meaning that acute coronary syndrome was lower on the differential.

Transthoracic echocardiogram was an invaluable tool during the pandemic because of its easy access, noninvasive nature, reliability, and feasibility. Our initial findings were a severely decreased left ventricular systolic function with an estimated ejection fraction of 20%, normal right ventricular systolic function, local segmented wall abnormalities, central venous pressure of 15 mmHg, and a small pericardial effusion.^1^ Regional segmented wall abnormalities were an akinetic inferolateral and anterior apical wall and hypokinetic basal and septal walls.^1^ Classic ECG findings of Takotsubo cardiomyopathy (eg, apical ballooning and basal wall hyperkinesis) were not apparent in this case.^3^ Moreover, the presence of pericardial effusion and pericarditis is also not typical of stress-induced cardiomyopathy. Also, note that the diagnosis of Takotsubo syndrome requires the exclusion of myocarditis. Again, we recognize that Takotsubo cardiomyopathy cannot be entirely ruled out; however, the presence of significant viral prodrome, elevated inflammatory markers, elevated cardiac markers, ECG findings, and pericardial effusion made the diagnosis of myopericarditis more likely.

We agree that endomyocardial biopsy would have confirmed the diagnosis of myocarditis. Unfortunately, an invasive procedure such as an endomyocardial biopsy was not feasible in the setting of hemodynamic instability, severe COVID-19 infection, and recent use of anticoagulants. Furthermore, according to the American College of Cardiology/American Heart Association, the class 1 indications for endocardial biopsy are (1) new-onset unexplained heart failure less than 2 weeks’ duration with hemodynamic compromise and (2) new-onset unexplained heart failure within 2 weeks to 3 months associated with left ventricle dilation and brady/tachyarrhythmias and failure to respond to standard care within 1 to 2 weeks.^4^ By hospital day 9, our patient had almost complete recovery of her left ventricular function with an estimated ejection fraction of 50%; therefore, an endomyocardial biopsy was not indicated.

The decision to use colchicine in this patient was reasonable given evidence of its efficacy in patients with pericarditis.^5^ At the time this case report was written, there were no clinical trials on the effectiveness of colchicine in COVID-19-associated myocarditis.

In conclusion, we do acknowledge that a lot has changed about our understanding of COVID-19; however, our hope is that this case report highlights the diagnostic and therapeutic challenges in the management of cardiac injury during the novel COVID-19 pandemic.
